# Supplementary material for: Empirical methods for controlling false positives and estimating confidence in ChIP-Seq peaks
Source: BMC Bioinformatics. 2008 Dec 5;9:523. doi: 10.1186/1471-2105-9-523 (PMC2628906; doi:10.1186/1471-2105-9-523)
Supplement: Additional File 1 — A variety of html documents from the USeq web site detailing the available applications, their best usage, output file type descriptions, command line menus, etc. [file 1471-2105-9-523-S1.zip › USeqUserGuides/JavaDoc.zip!/JavaDoc/util/bio/wrappers/package-frame.html]

util.bio.wrappers


util.bio.wrappers

Classes 
  
BL2Seq
  
BL2SeqHit
  
Blast
  
BlastResult
  
Primer3Pick
  
Primer3Wrapper
  
Segment
  
SimpleBlast
